# A Kenyan perspective on the use of animals in science education and scientific research in Africa and prospects for improvement

**DOI:** 10.4314/pamj.v9i1.71224

**Published:** 2011-08-25

**Authors:** Charles Kimwele, Duncan Matheka, Hope Ferdowsian

**Affiliations:** 1 Department of Veterinary Anatomy and Physiology, University of Nairobi, Kenya; 2 Africa Network for Animal Welfare, Nairobi, Kenya; 3 Department of Medical Physiology, University of Nairobi, Kenya; 4 Physicians Committee for Responsible Medicine, Washington, D.C, USA; 5 Department of Medicine, The George Washington University

**Keywords:** Animal Research, Experimentation, Education, Three Rs, Alternatives, Ethics, Kenya, Africa

## Abstract

**Introduction:**

Animal experimentation is common in Africa, a region that accords little priority on animal protection in comparison to economic and social development. The current study aimed at investigating the prevalence of animal experimentation in Kenya, and to review shortfalls in policy, legislation, implementation and enforcement that result in inadequate animal care in Kenya and other African nations.

**Methods:**

Data was collected using questionnaires, administered at 39 highly ranked academic and research institutions aiming to identify those that used animals, their sources of animals, and application of the three Rs. Perceived challenges to the use of non-animal alternatives and common methods of euthanasia were also queried. Data was analyzed using Epidata, SPSS 16.0 and Microsoft Excel.

**Results:**

Thirty-eight (97.4%) of thirty-nine institutions reported using animals for education and/or research. Thirty (76.9%) institutions reported using analgesics or anesthetics on a regular basis. Thirteen (33.3%) institutions regularly used statistical methods to minimize the use of animals. Overall, sixteen (41.0%) institutions explored the use of alternatives to animals such as cell cultures and computer simulation techniques, with one (2.6%) academic institution having completely replaced animals with computer modeling, manikins and visual illustrations. The commonest form of euthanasia employed was chloroform administration, reportedly in fourteen (29.8%) of 47 total methods (some institutions used more than one method). Twenty-eight (71.8%) institutions had no designated ethics committee to review or monitor protocols using animals.

**Conclusion:**

Animals are commonly used in academic and research institutions in Kenya. The relative lack of ethical guidance and oversight regarding the use of animals in research and education presents significant concerns.

## Introduction

Studies have demonstrated that animals experience significant pain and distress as a result of their use in experimentation [1–5]. For example, even in response to being handled, animals experience marked changes in physiological and hormonal markers of stress [6]. Scientific advancements regarding animals’ cognitive and emotional capacities should be reflected in the development and refinement of guidelines regarding their use in research.

Currently, there is scarce empirical data from Africa on the existence or adequacy of national or institutional policies and guidelines on the use of animals in research. However, the little evidence available indicates that most African countries lack relevant legislation and guidelines [7]. Paradoxically, a rise in the use of animals for research and education has been observed.

Most countries that were under British influence have animal protection laws derived from Britain's Protection of Animals Act of 1911 [8]. Many other African nations lack published animal welfare laws. Some countries have enacted their own animal welfare legislation [9-13], but even in these cases, implementation and enforcement mechanisms have been largely ineffective. These countries include Kenya [9], South Africa [10,11,13], and Tanzania, which enacted the Animal Welfare Act in 2008 [12].

In Tanzania, researchers must obtain a permit issued by the relevant minister or by the Director of Animal Welfare under advice of the Animal Welfare Council. Some guidelines are also provided, such as the need for veterinary supervision. However, implementation has been delayed, and the various statutory organs stipulated in the Act have yet to be established. In South Africa, research is guided by the South Africa Medical Research Council Act, No. 58 (1991) [11], which developed internal guidelines on the use of animals in research in 2004 [13]. In Kenya, all vertebrates are protected under Cap 360 (the Prevention of Cruelty to Animals Act) (1963, revised 1983). However, enforcement remains a challenge. Although Tanzania laws cover all animals, Kenya and South Africa animal research laws only cover vertebrates, despite studies having indicated that vertebrates and invertebrates can display signs of pain or distress [4,5].

It is possible that some African countries may have developed guidelines that are not available in the public domain and are therefore not known by the authors and generally unknown to the larger scientific community.

In order to better understand some general trends regarding the use of animals in research and education in Africa, we collected and analyzed preliminary data from various institutions in Kenya, one of the leading countries in sociopolitical and economic development in Africa.

## Methods

Structured and semi-structured questionnaires were administered at 39 highly ranked academic and research institutions in Kenya [14]. Of these, 30 were academic institutions and departments whereas nine were non-academic research institutions. Surveys were completed by senior personnel within departments directly involved in scientific research and education. The questionnaire, administered in August 2010, was developed to collect data for the preceding year 2009. The study was aimed at determining whether the institutions used animals in research or education; what species they used; the number of animals used for each species; whether institutions incorporated alternatives to the use of animals, as suggested by the three Rs framework developed by Russell and Burch (Reduction, Refinement and Replacement) [15]; and whether there were perceived obstacles to the incorporation of alternatives to the use of live animals in research and education. The survey also included questions about the sources of animals, euthanasia techniques, and the presence or absence of ethical review committees within each institution. Ethical approval for the study was granted by The University of Nairobi's Biosafety, Animal Use and Ethics committee. Respondents were assured that no participating institution would be identified, and no personal information was collected for the purposes of the study. The data was analyzed using Epidata (Odense M, Denmark), SPSS version 16.0 (Chicago, Illinois, USA) and Microsoft Excel (Redmond, Washington, USA) and presented as descriptive data.

## Results

### The prevalence of animal use in education and research in Kenya

**Figure 1:**
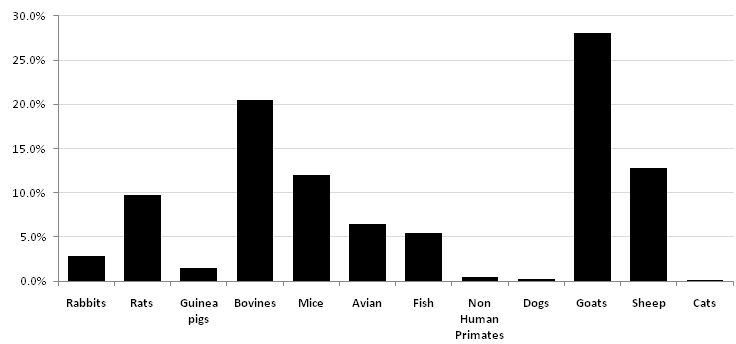
Percentage of animals commonly used in Kenya for research and education

Of the 39 institutions surveyed, 38 (97.4%) reported use of animals in research and/or education. The animals used included mammalian, avian and fish species (Figure 1). According to survey results, we estimated that an annual total of 35,284 animals were used. Of the total animals used, caprines accounted for 28.0% (n=9883, mean 299.5, range 0-9,792) of the animals used; bovines, 20.5% (n=7,234, mean 219.2, range 0-6,674); ovines, 12.8% (n=4,516, mean 136.8, range 0-4,288); mice, 12.0% (n=4,244, mean 128.6, range 0-1,000); rats, 9.7% (n=3,427, mean 103.8, range 0-750); chickens, 6.4% (n=2,260, mean 68.5, range 0-1,835); fish 5.5% (n=1,926, mean 58.4, range 0-1,920); rabbits, 2.9% (1,023, mean 31.0, range 0-200); guinea pigs, 1.5% (n=535, mean 16.2, range 0-110); nonhuman primates, 0.4% (n=150, mean 3.8, range 0-150); dogs, 0.2% (n=71, mean 2.15, range 0-61); and cats, 0.04% (n=15, mean 0.45, range 0-15). Thirteen (33.3%) institutions reported regularly using statistical methods to minimize the use of animals.

### Sources of animals

Respondents reported different sources of animals. These sources were categorized as academic or research institution breeding facilities (76.8%), farms (12.2%), commercial breeding operations (7.4%), animals’ natural habitats (2.4%), and local council animal control facilities (1.2%).

### Alternatives to animal use in research and education

Sixteen (41.0%) institutions used alternatives to the use of live animals, most commonly animal cell cultures (n=16) and computer simulators (n=10). Only one institution used human cell cultures as a replacement to live animal use. In all, thirty-six (92.3%) respondents cited financial constraints as a major obstacle to the use of alternatives in research and education.

### Analgesia and euthanasia

Only 30 (76.9%) of 39 institutions reported using analgesics or anesthetics on a regular basis. According to the survey, 18.0% (n=7) of institutions had no clear euthanasia methods and a further 5.1% (n=2) of institutions did not euthanize the animals at end-point.

Experimental end-point euthanasia varied across institutions. A total of 47 methods of euthanasia were reported. According to survey responses, inhalant chloroform and diethylether were most commonly used for euthanasia at 29.8% (n=14 of 47 methods) and 21.3% (n=10 of 47 methods), respectively. Other reported methods included cervical dislocation, barbiturate overdose and slaughter (either using a captive bolt for stunning followed by slitting the throat to incise major blood vessels for the large livestock, or only slitting of the throat for smaller livestock used in research) at 8.5% (n=4 of 47 methods) each, and decapitation and embolism at 2.1% (n=1 of 47 methods) each.

### Ethical review and oversight

Twenty-eight (71.8%) institutions had no designated committee to review or monitor protocols using animals. Only two of the institutions with an established animal care and use committee referred to documented guidelines, and neither documented the composition of their committees.

## Discussion

We identified several issues that raise serious ethical and welfare concerns regarding the use of animals in education and research in Kenya. The majority of surveyed institutions reported using animals in research or education. Similar to other regions of the world, Kenyan researchers use animals for basic research, antibody development and production, and pharmacology and toxicology studies. Undergraduate veterinary and medical education facilities use animal dissections for anatomical illustrations. Organs are isolated and used for physiological demonstrations, and whole animals are used for demonstrating surgical techniques [16].

The majority of institutions did not have established committees to review experimental protocols or to provide oversight regarding the use of animals in educational and research settings. Only two of the institutions with an established animal care and use committee referred to documented guidelines, one of which made reference to external guidelines, specifically the United States National Research Council's Guide for the Care and Use of Laboratory Animals [17] and the United Kingdom Animals (Scientific Procedures) Act 1986 [18].

The internationally accepted three Rs framework (Reduction, Refinement and Replacement) forms a useful, albeit limited, guide for promoting animal welfare [15, 19,20]. These standards, which were first published in The Principles of Humane Experimental Technique by William Russell and Rex Burch in 1959, sensitize researchers to reduce the number of animals used, refine their techniques so as to limit the pain and distress animals experience, and finally to replace the use of animals with non-animal alternatives wherever possible [15, 20]. Such alternatives are widely available, and their development and refinement has of late intensified. Institutional animal care and use committees commonly use the three Rs as a guide in assessing and monitoring animal use projects in their institutions. However, our survey found that these committees were rare across Kenyan academic and research institutions. More than seventy percent of the institutions had no animal care and use committee, despite their use of animals in education and research.

Based on survey results, there are several other areas that deserve further examination. Reported methods of euthanasia raised serious welfare concerns. For example, the use of chloroform has been criticized based on concerns about human and animal welfare. Chloroform is associated with significant hepatotoxicity and carcinogenicity [21].

Although the questionnaire did not cover questions about housing or husbandry standards, this is another area which deserves further investigation. Currently, there are no regulations that require monitoring of animal breeding facilities in Kenya, as this is not covered in the Prevention of Cruelty to Animals Act [9].

Policies, legislative frameworks and guidelines on the use of animals are all poorly developed in Africa. In Kenya, the Prevention of Cruelty to Animals Act (1963, revised in 1983) requires researchers to obtain a permit to conduct research from the minister for livestock development, for which justification must be made to include one of the following aims: (a) advancement of human and animal health, (b) discovery of new scientific knowledge, or (c) testing of an earlier discovery [9]. There is also an unenforced requirement that animal experiments must be conducted under anesthesia, with undefined exemptions. There are no requirements for protocol review by an ethics committee. Finally, this Act has no enforcement mechanism.

There are increasing efforts to improve global standards for animal welfare, which may offer opportunities for improvement within Africa. For example, statements on assuring the welfare of animals are required for most peer-reviewed journals in the life sciences [22, 23]. The Animal Research: Reporting In Vivo Experiments (ARRIVE) guidelines, intended to improve the reporting of animal experiments, also offer opportunities to enhance transparency in protocol review and compliance with national or institutional guidelines for the care and use of animals [22].

Additionally, the Office International Epizooties (OIE) of the World Organization for Animal Health, with 176 member states, has set standards for the transport and slaughter of animals. Currently, OIE is seeking to expand its mandate to cover all aspects of animal welfare. OIE's guiding principles state, inter alia, that the internationally recognized three Rs provide valuable guidance for the use of animals in science, and that the use of animals carries with it an ethical responsibility to ensure their welfare to the greatest extent possible [24].

A proposed Universal Declaration on Animal Welfare [25] seeks an agreement among people and nations to recognize that animals are sentient beings and can suffer, to respect their welfare needs, and to end animal cruelty. Introduced in 2003 at an international meeting in Costa Rica and currently endorsed by five countries, this declaration is awaiting adoption by the UN General Assembly and subsequent ratification. African animal welfare organizations from 28 countries have supported this initiative through a proposed African Declaration of Animal Welfare, and it is pending adoption by the African Union.

The absence of legal and ethical frameworks and committees to review protocols that involve animals in research and education leaves major gaps in the protection of the animals involved. In addition, the lack of institutional animal ethics committees promotes the outsourcing of animal research to these unregulated institutions [7].

Unfortunately, the increasing use of animals in education and research is not matched by the development of regulatory frameworks to promote ethical practice that guide scientific investigation using animals. The relative lack of funding, among other challenges, is a perceived obstacle to the use of alternative techniques. This scenario, coupled with the need to train students as well as the desire for personal advancement in the academic and scientific arena, has resulted in the use of one of the most readily available and vulnerable resources-animals. It is also critical for researchers to consider how an experiment on animals can be replaced, not only by cell cultures or computer models, but also by what is almost certainly more important—ethically conducted studies of human disease, whether in patients, tissues, or populations. Such studies are not only ethical; they sidestep the problems of extrapolating from animals to humans. There is therefore a need to establish training programs for educators, researchers, and policy makers regarding the scientific and ethical considerations regarding the use of animals in education and research.

## Conclusion

Animals are commonly used in academic and research institutions in Kenya. The use of alternatives to animals in research and education was rarely reported. Further, the majority of institutions surveyed did not have ethical review committees established to review experimental protocols. Our preliminary findings emphasize the need for establishment and enforcement of strict policies and guidelines regarding the use of animals in research and education in Kenya and perhaps elsewhere in Africa.
